# Uneven sequencing (coverage) depth can bias microbial intraspecies diversity estimates and how to account for it

**DOI:** 10.1093/ismeco/ycaf228

**Published:** 2025-12-06

**Authors:** Esteban Bustos-Caparros, Tomeu Viver, Juan F Gago, Stephanus N Venter, Rafael Bosch, Konstantinos T Konstantinidis, Luis M Rodriguez-R, Ramon Rossello-Mora

**Affiliations:** Marine Microbiology Group (MMG), Department of Animal and Microbial Biodiversity, Mediterranean Institute for Advanced Studies (IMEDEA, CSIC-UIB), 07190 Esporles, Spain; Marine Microbiology Group (MMG), Department of Animal and Microbial Biodiversity, Mediterranean Institute for Advanced Studies (IMEDEA, CSIC-UIB), 07190 Esporles, Spain; Marine Microbiology Group (MMG), Department of Animal and Microbial Biodiversity, Mediterranean Institute for Advanced Studies (IMEDEA, CSIC-UIB), 07190 Esporles, Spain; Department of Biochemistry, Genetics and Microbiology, and Forestry and Agricultural Biotechnology Institute (FABI), University of Pretoria, 0002 Pretoria, South Africa; Microbiologia, Departament de Biologia, Edifici Guillem Colom, Universitat de les Illes Balears, Campus UIB, 07122 Palma de Mallorca, Spain; School of Civil and Environmental Engineering and School of Biological Sciences, Georgia Institute of Technology, 3033 Atlanta, Georgia, United States; Department of Chemistry and Biosciences, Aalborg University, 9220 Aalborg, Denmark; Department of Microbiology and Digital Science Center (DiSC), University of Innsbruck, 6020 Innsbruck, Austria; Marine Microbiology Group (MMG), Department of Animal and Microbial Biodiversity, Mediterranean Institute for Advanced Studies (IMEDEA, CSIC-UIB), 07190 Esporles, Spain

**Keywords:** sequencing depth, nucleotide diversity, ANIr, metagenomics, bias

## Abstract

An unbiased and accurate estimation of intraspecies diversity, i.e. the extent of genetic diversity within species (or microdiversity), is crucial for clinical and environmental microbiome studies. Although it is well appreciated that sequencing depth (or coverage depth) below 10X can provide biased estimates of microdiversity, typically underestimating diversity due to the random sampling of alleles, there is a widely accepted convention that microdiversity estimates tend to be relatively stable at sequencing depth exceeding 10X. Therefore, discarding species with <10X or rarefying to 10–20X sequencing depth are generally used to compare microdiversity among taxa and samples. Our findings showed that these biases may persist even at depth levels above 50-200X for all popular sequencing platforms, including Illumina, PacBio, and Oxford Nanopore. The biases mostly, but not always, represent an underestimation of diversity and were attributable to the incomplete recovery of Single Nucleotide Variants (SNVs) at lower sequencing depth levels. To address this issue, we recommend using rarefaction-based approaches to standardize data at least 50X, and ideally at 200X sequencing depth, which reduces differences between observed and expected microdiversity values to <0.5%. Furthermore, the Average Nucleotide Identity of reads (ANIr) metric is significantly less sensitive to sequencing depth variability than nucleotide diversity (*π*), making it a robust alternative for estimating microdiversity at sequencing depth close or exceeding 10X, without a need to rarefying data. Therefore, the sequencing depth thresholds proposed herein provide a more standardized framework for direct comparisons of microdiversity across samples and studies.

## Introduction

Intraspecies diversity, often referred to as microdiversity, describes the genetic heterogeneity within a microbial species (i.e. the number and diversity of coexisting strains) [1–12]. Unlike community alpha-diversity metrics such as Shannon *H′* [13] or Nonpareil *N_d_* [14], which quantify species-level diversity within microbial communities, microdiversity focuses exclusively on variations within a single species [1–12]. With the advent of metagenomics, allowing the sequencing of (almost) entire microbial communities, there has been growing interest in understanding microdiversity patterns and dynamics given the recognition that intraspecies diversity can impact ecological resilience, microbial adaptation, and host-associated functions, including human health [1–12].

Among the most widely used metrics to quantify microdiversity in metagenome-based studies are nucleotide diversity (*π*) [1, 5, 15–17] and the Average Nucleotide Identity of reads (ANIr) [18–22]. *π* is calculated as the sum of squared nucleotide frequencies at each site: *π* = 1 − [(frequency of A)^2^ + (frequency of C)^2^ + (frequency of G)^2^ + (frequency of T)^2^] [5]. Precisely, a given site with no Single Nucleotide Variants (SNVs), where only one nucleotide is present at frequency 1, yields a *π* value of 0. In contrast, a site with all four nucleotides present at equal frequency (0.25) yields a *π* estimate of 0.75. After calculating *π* values at each site, the average across the genome gives the genome-wide *π* estimate [5]. Instead, ANIr reflects the average nucleotide identity of metagenomic reads mapped to a Metagenome-Assembled Genome (MAG) or isolate genome [2, 18–22]. ANIr values generally range from 95% to 100% (i.e. the species genomic boundary), where higher values indicate more homogeneous populations (i.e. low microdiversity) and lower values reflect greater intraspecific heterogeneity (i.e. high microdiversity) [2, 18–22]. As MAGs represent the consensus assemblies of multiple coexisting strains of a given species [2, 3, 8, 11], shifts in *π* and ANIr estimates are used as proxies for estimating changes in strain composition along time- or spatial-series [1–22].

Several studies have shown that the accuracy of both *π* and ANIr is influenced by two sequencing-related metrics: (i) sequencing depth, or coverage depth, and (ii) sequencing breadth, or coverage breadth [1, 2, 5, 15, 16]. Specifically, sequencing depth quantifies the average number of reads mapping at each base of a reference sequence such as a genome or gene. That is, a sequencing depth of 10X means each base is covered, on average, by 10 reads. Instead, sequencing breadth measures the percentage of the reference sequence that is mapped by reads, with a 25% sequencing breadth meaning that a quarter of the reference sequence is covered by metagenomic reads. For both metrics, it has been extensively observed that low sequencing depth, especially below the 5–10X range, negatively impacted the accuracy of *π* and ANIr estimates [1, 5, 15]. At low depth, SNV detection, which directly influences *π* and ANIr values, becomes increasingly unreliable due to stochastic read sampling, leading to under- or overestimation of microdiversity [5, 15]. Furthermore, sequencing breadth below 50% has also been described as potentially introducing bias to *π* values [5]. Nevertheless, it has been described that estimates of *π* and ANIr apparently tend to stabilize beyond 10X sequencing depth [2, 5, 15], resulting in the wide acceptance of 10X sequencing depth as a threshold for many microdiversity studies using metagenomics [1, 2, 5, 15–17].

Despite the 10X sequencing depth practical threshold, there is no standardized framework for handling microdiversity estimation using *π* and ANIr, which potentially result in systematic biases on microdiversity studies. In general, two main and distinct strategies are generally employed: (i) exclude MAGs or isolate genomes with sequencing depth below 5–10X from further analysis and subsequently compare taxa regardless of the variability in sequencing depth among samples [11, 23–27], or (ii) rarefying, meaning down-sampling reads to fixed thresholds, to typically 5X, 10X, or 20X sequencing depth, to normalize any effects of uneven sequencing depth on diversity metrics [1, 5, 15, 17, 22, 28–30]. Here, we aim to characterize the effects of these interventions to guide objective, data-driven standardization procedures that would enable the robust comparison of microdiversity measured in metagenomic datasets across samples.

Recent studies focused on marine [31], wastewater [31], and human gut [32] microbiomes have revealed that metagenomes with lower sequencing depth resulted in lower observed community richness (i.e. fewer species detected), compromising the detection of taxa per sample [31, 32]. Therefore, considering the large intraspecies diversity described in both clinical and environmental settings, with potentially hundreds to thousands of distinct strains per species in a single site [4–8, 10–12], we hypothesized that *π* and ANIr may still be unreliably estimated even at sequencing depth above 10X, meaning that the full-extent of intraspecies diversity of a given species cannot be recovered at this level. Consequently, practices such as rarefying or applying fixed cutoffs to relatively low sequencing depth levels (e.g. 10–20X) may bias biological differences in terms of intraspecies diversity.

To test this hypothesis, affecting diversity comparison across datasets and studies, we assessed the impact of sequencing depth and sequencing breadth on *π* and ANIr estimates using a combination of environmental and synthetic metagenomes. In order to assess the effects of sequencing errors on diversity estimates, we generated in parallel 36 synthetic metagenomes using defined mixtures of *Salinibacter ruber* strains comprising from 1 up to 100 genomes and pairwise average nucleotide identity (ANI) values between 95% and 100%. These mock communities were simulated under three sequencing error profiles: error-free, Q20, and Q30 reads, resulting in 12 metagenomes per each profile, which would allow us to disentangle the individual effects of strain-level diversity, read quality or sequencing error, and sequencing depth on microdiversity estimates. The environmental collection comprised 53 publicly available metagenomes derived from three distinct environments: hypersaline ecosystems [21], marine waters [33, 34], and the human gut [35], generated with Illumina, PacBio, or Oxford Nanopore technologies. From each environment, we selected the 10 most abundant MAGs (i.e. 30 in total) as focal taxa. This environmental dataset would be used to corroborate the findings observed using the simulated dataset.

## Materials and methods

### Short- and long-read metagenome processing

Metagenomes and MAGs used in this study were summarized in Supplementary Tables S1 and S2, respectively. Material and methods used to evaluate the effects of sequencing depth on *π* and ANIr estimates were summarized in Supplementary Fig. S1. Specifically, Illumina raw reads were trimmed using bbduk v38.82 (https://sourceforge.net/projects/bbmap/; quality score ≥20 and length ≥100 bp). Next, trimmed Illumina reads and PacBio or Oxford Nanopore (ONT) long-reads were randomly subsampled selecting the 1%, 5%, 10%, 20%, 30%, 40%, 50%, 60%, 70%, 80%, and 90% of the total reads using the *FastA.subsample.pl* script from enveomics collection [36]. To directly compare short- vs long-reads for their accuracy in microdiversity estimates, we fragmented long reads in 200 bp fragments using the script *shred.sh* (https://sourceforge.net/projects/bbmap/). Trimmed reads were mapped to the contigs of MAGs using Bowtie2 v2.3.4.1 [37], and best-match mapped reads were filtered at 95% identity with samtools v.1.10 and bedtools v.2.30.0 [38, 39].

### *In silico* metagenome generation and processing

From our collection of *Sal. ruber* isolate genomes listed in Supplementary Table S3, we selected 1, 5, 10, 20, 30, 40, 50, 60, 70, 80, 90, and 100 genomes for *in silico* metagenome simulations (Supplementary Table S4) using scripts available at: https://github.com/baldeguer-riquelme/Nonpareil-sequencing-standardization [31]. Relative abundances of each genome in all *in silico* metagenomes followed a log-normal distribution. For no-error, Q20, and Q30 metagenomes we used: *python MetaG_simulator.py illumina --genome list.txt --num_sp 100 --num_genomes_per_sp 1 --num_metagenomes 1 --t 16 --metag_size 10 000 000* with the additional parameters: *--out Noerror --error_prob “0.0,0.0,0.0,0.0,0.0”* (no-error); *--out Q20 --error_prob “0.001,0.001,0.006,0.001,0.001”* (Q20); and *--out Q30 --error_prob “0.0001,0.0001,0.0006,0.0001,0.0001”* (Q30). Numbers separated by commas in the *--error_prob* parameter indicated the probability of each read having an insertion, deletion, mismatch, mismatch at the beginning and at the end of the read, respectively. Similar to environmental metagenomes, each *in silico* metagenome was randomly subsampled selecting the 1%, 5%, 10%, 20%, 30%, 40%, 50%, 60%, 70%, 80%, and 90% of the total reads using the *FastA.subsample.pl* script from enveomics collection [36].

### Nucleotide diversity (π), ANIr, and sequencing depth estimation

Sequencing depth, nucleotide diversity (*π*), and ANIr were estimated with inStrain v1.5.4 [5] using the “profile” operation and the following parameters: *--pairing_filter non_discordant --skip_mm_profiling --min_cov 5 --min_read_ani 0.95*. Variables affecting *π* were evaluated using ADONIS [40] as implemented in the function adonis2 from the R package vegan v2.7–1 [41] with a formula placing differences in *π* in the left hand side and the following variables additively, where relevant and in that order, in the right hand side: species, sequencing effort (in base pairs, log-transformed, and center-scaled), average PHRED score (center-scaled), environmental variables (salinity and temperature, centered-scaled), and sequencing depth (log-transformed and centered-scaled). Significance was assessed sequentially (by = “terms”) using 999 permutations and 8 threads.

## Results and discussion

### Uneven sequencing depth can bias nucleotide diversity (*π*) estimates

**Figure 1:**
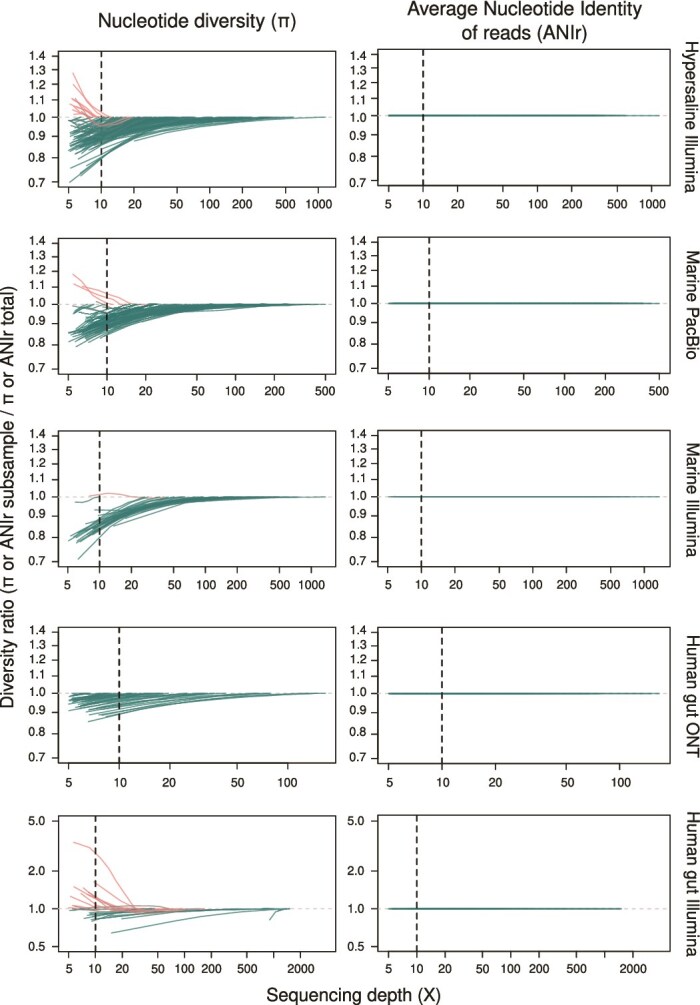
Influence of increasing sequencing depth on nucleotide diversity (*π*) and ANIr estimates. Estimation of the impact of sequencing depth on the accuracy of nucleotide diversity (*π*; left panels) and Average Nucleotide Identity of reads (ANIr) (right panels) estimates across hypersaline, marine or human gut metagenomes using Illumina, PacBio, or Oxford nanopore (ONT) sequencing platforms. The microdiversity ratio represents the *π* or ANIr obtained for each subsample of a metagenome divided by the *π* or ANIr of the whole-metagenome, with ratio = 1 meaning that microdiversity in the subsample is equal to microdiversity in the whole-metagenome. Note that each line represents the microdiversity estimate of one MAG in a given metagenome. Green lines indicated larger microdiversity at increasing sequencing depth (i.e. expected trend; microdiversity ratio <1) and red lines indicated larger microdiversity at lower sequencing depth (i.e. microdiversity ratio >1). Dashed lines indicate a sequencing depth of 10X.

**Figure 2:**
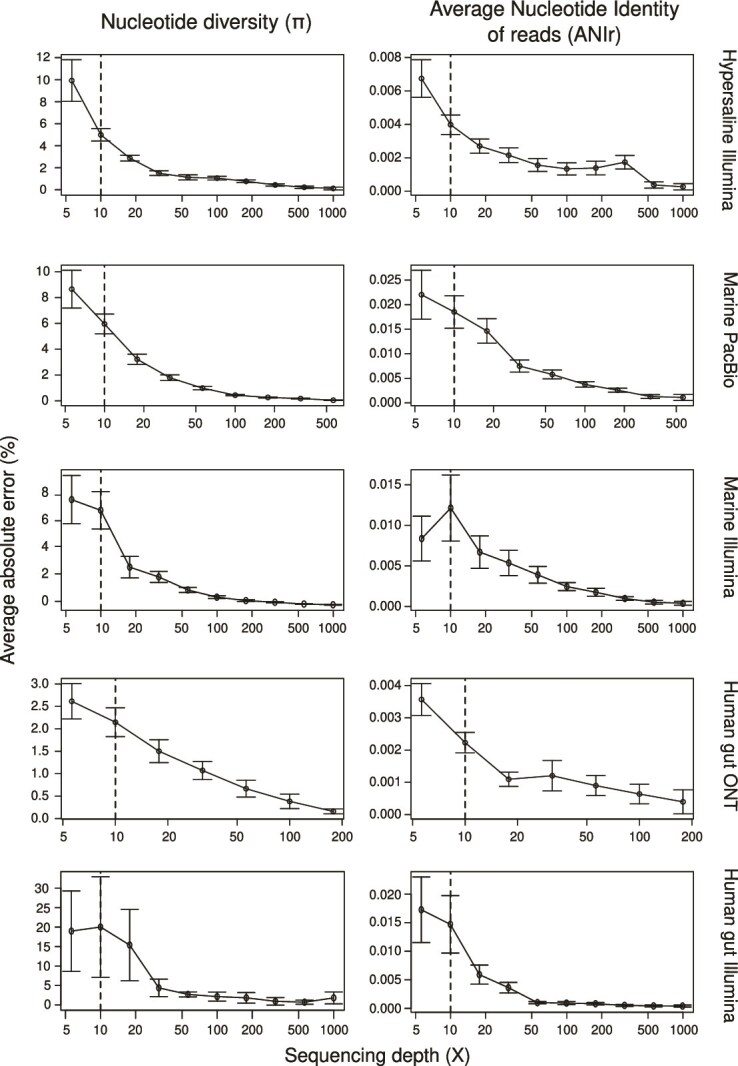
ANIr estimates show lower average absolute errors (%) than nucleotide diversity (*π*) at equal sequencing depth. Estimation of the average absolute error (%) and standard deviation of nucleotide diversity (*π*; left panels) and Average Nucleotide Identity of reads (ANIr) (right panels) estimates across increasing sequencing depth along hypersaline, marine, or human gut metagenomes using Illumina, PacBio, or Oxford nanopore (ONT) sequencing platforms. The error is calculated as the difference between the *π* or ANIr estimates for each subsampled fraction of the metagenome and the estimate for the full-size metagenome before subsampling. Dashed lines indicate a sequencing depth of 10X.

We first aimed to investigate the effect of sequencing depth on metagenomic estimates of *π* in order to determine to which extent sequencing effort can affect this metric. We do not consider here sequencing breadth because an estimated depth of 5X is already predicted to reach a breadth in excess of 99% [42], so nearly complete coverage breadth is expected at the levels of sequencing effort investigated here. However, we note that a breadth of 50% is often used as a filter in the literature, which would be far from sufficient. We evaluated this effect by devising the metric of diversity ratio, which denotes the value of *π* estimated from each subsampled dataset relative to that value obtained from the full dataset (Supplementary Fig. S2). For all sequencing platforms and environments, trends of diversity ratio showed two distinct regimes. At sequencing depth below 10X, *π* estimates were highly variable, occasionally overestimating *π* (i.e. larger *π* in subsampled than full dataset; diversity ratio >1; red lines), which strongly aligned with the potential stochastic detection of rare alleles at sequencing depth below 10X reported previously [1, 5], but more often underestimating (i.e. lower *π* in subsampled dataset; diversity ratio < 1; green lines) *π* (Fig. 1). According to these trends, below 10X sequencing depth we detected larger absolute average error across sequencing platforms and environments, which ranged from 2.5% up to 30% (Fig. 2). Conversely, at sequencing depth exceeding 10X the overestimation (i.e. red lines) of *π* was reduced across environments and sequencing platforms (Fig. 1) and the divergence (i.e. error) between subsampled and full datasets decreased (Figs 1 and 2). These findings were consistent with prior reports indicating that sequencing depth >10X substantially improves microdiversity estimation accuracy [1, 5]. Despite this increase in accuracy, however, we observed that beyond 10X, underestimation (i.e. diversity ratio < 1; green lines) remained largely constant (Figs 1 and 2), suggesting that a substantial fraction of microdiversity remained undetected above 10X sequencing depth.

As sequencing depth increased, differences between *π* estimates at subsampled and full datasets were notably reduced, with the diversity ratio being >0.9 at sequencing depth higher than 50X and >0.95 at 200X (Figs 1 and 2). However, the diversity ratio did not plateau even at sequencing depth exceeding 1400X (Fig. 1; Supplementary Fig. S3), indicating that high sequencing depth may be required to capture the full extent of intraspecies diversity. This finding is also consistent with previous reports that novel SNVs were still detected at ~500X [1]. These findings are in line with numerous studies reporting extensive microdiversity in both environmental and clinical settings [1, 4–22], including a recent observation showing the coexistence of >11 000 distinct genomovars within a single species, *Sal. ruber*, at a single saltern site [12]. Moreover, our results further highlighted that even deeply sequenced metagenomes, such as those from the human gut and marine ecosystems, often fail to recover the full extent of intraspecies diversity.

### Sequencing depth below 50X produce spuriously variable nucleotide diversity (*π*) estimates

**Figure 3:**
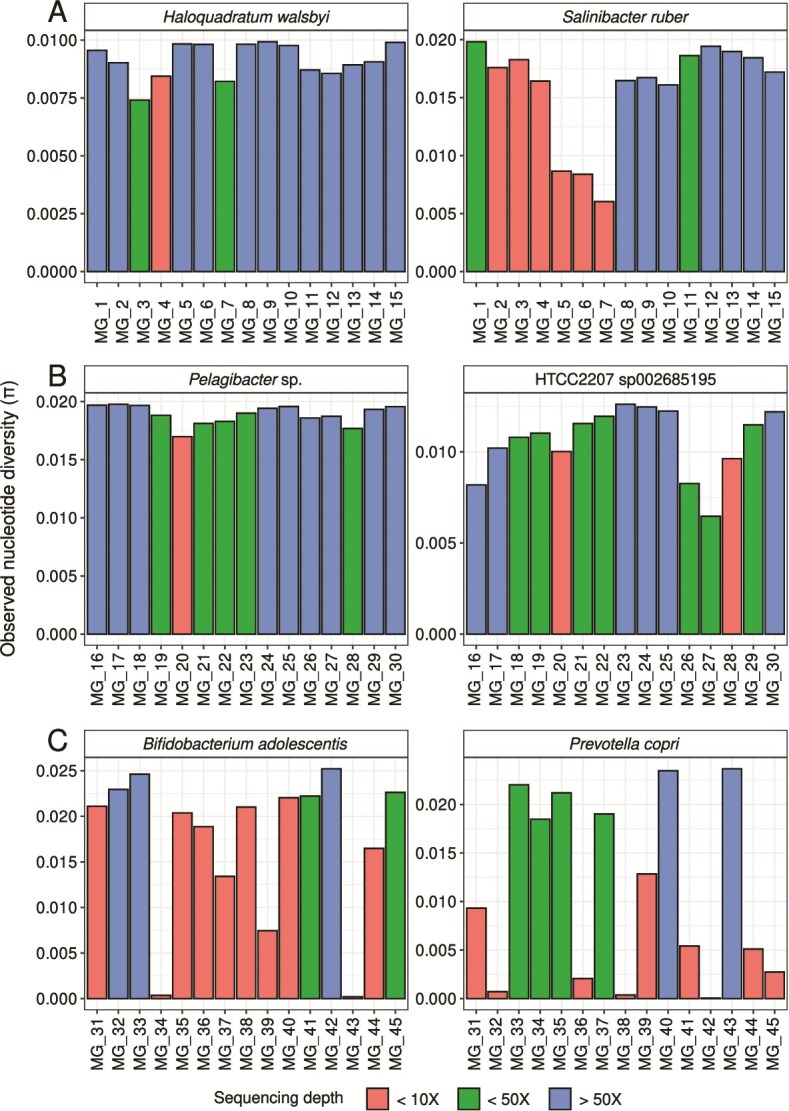
Differences in nucleotide diversity (*π*) estimates were reduced at sequencing depth above 50X. Barplots showing the comparison of nucleotide diversity (*π*) estimated using the full-size metagenome (i.e. observed) of two relevant taxa in (A) hypersaline, (B) marine, and (C) human gut metagenomes. Color gradient shows the sequencing depth categories for each species in a given metagenome.

To further investigate the ecological relevance of comparing *π* estimates across metagenomes with uneven sequencing depth, we assessed the differences in *π* estimates using the whole-metagenome (i.e. observed estimates) across metagenomes. We first evaluated a hypersaline collection, which comprised a set of 15 metagenomes of a time-series experiment where recurrent osmotic disturbances were applied over 2.5 years [21]. By focusing on the two most widespread and relevant taxa in hypersaline environments, *Haloquadratum walsbyi* [43] and *Sal. ruber* [44, 45], we found the largest discrepancies in *π* estimates (either low or high *π* values) along metagenomes at sequencing depth below 10X for both species, followed by estimates at sequencing depth below 50X (Fig. 3A). As expected, based on the abovementioned results, differences between *π* estimates across metagenomes were remarkably reduced at sequencing depth larger than 50X for both species (Fig. 3A). For example, direct comparisons (i.e. without normalizing) of *π* estimates with uneven sequencing depth would suggest that *Hqr. walsbyi* in the sample MG_3 (sequencing depth = 17.1X; *π* = 0.007) was less diverse than in MG_15 (sequencing depth = 329.7X; *π* = 0.009) (Fig. 3A). Similarly, direct comparisons among metagenomes for *Sal. ruber*, would lead to the interpretation that this species was more diverse in MG_6 (sequencing depth = 5.6X; *π* = 0.008) than in MG_12 (sequencing depth = 131.6X; *π* = 0.019) (Fig. 3A).

This observed trend was not exclusive of these two species; for almost all species studied here, the most discrepant *π* estimates were also detected in samples with sequencing depth below 50X (Fig. 3B and C, Supplementary Figs S4–S8). Focusing on two abundant species of the marine collection (*Pelagibacter* sp. and HTCC2207 sp002685195; [33, 34]), and two abundant species of the human gut microbiome (*Bifidobacterium adolescentis* and *Prevotella copri*; [35])*,* we also found that metagenomes in which these species had <50X, *π* estimates were generally lower than the observed values above 50X (Fig. 3B and C). The fact that variation in high-depth samples is much lower than low-depth samples suggest that sequencing depth, rather than natural variation, is chiefly responsible for the variance of observed values of *π*. To corroborate this observation, we performed an ADONIS analysis of the *π* values as a function of the species, technical features of the metagenome, environmental variables (for the hypersaline and marine collections), and sequencing depth. Even after accounting for other technically and biologically relevant variables, the sequencing depth had a significant impact on *π* in all three collections (*P* ≤ 0.0002).

Since we observed consistent patterns across taxonomically distant species of *Bacteria* and *Archaea* from distinct environments and sequencing platforms, our findings underscored the necessity of normalizing *π*-based diversity by sequencing depth to minimize potential biases in cross-dataset comparisons (e.g. time series or clinical trials). Accordingly, the typical practice of discarding data (e.g. species, genes) with <10–20X and comparing those above this threshold without normalization of sequencing depth [11, 23–27] or rarefying to sequencing depth close to 10-20X [1, 5, 15, 17, 22, 28–30] may provide inaccurate *π* estimates. Therefore, we recommend that for *π*-based comparisons, species would ideally require sequencing depth exceeding 50X. Further, rarefactions, if applied, might be carried out at sequencing depth ≥ 50X to prevent potential underestimation of intraspecies diversity and to preserve the accuracy of *π*-based microdiversity analyses.

**Figure 4:**
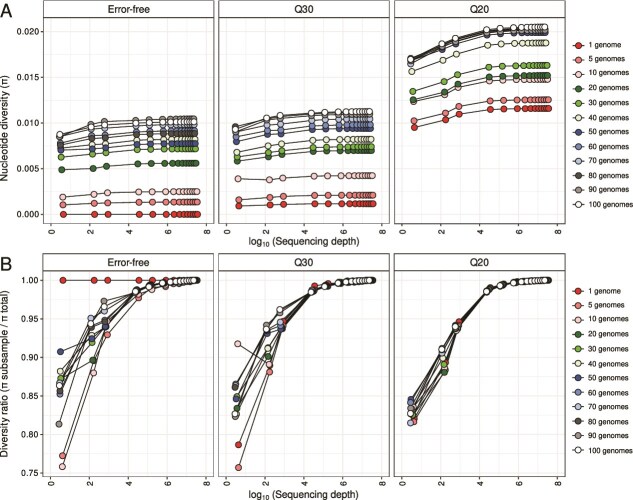
Sequencing errors and uneven sequencing depth impacts nucleotide diversity (*π*) estimates even within a clonal population. (A) Estimation of nucleotide diversity (*π*) values at increasing sequencing depth and isolate diversity on the 32 distinct *in silico* metagenomes with variable sequencing error profiles (i.e. error-free, Q20, Q30). Note that sequencing depth values are in represented logarithmic scale. (B) Estimation of the effect of increasing sequencing depth on the accuracy of nucleotide diversity (*π*) across *in silico* metagenomes with distinct proportions of isolate genomes and sequencing error profiles. The diversity ratio represents the estimated *π* at each subsample of a metagenome divided by *π* estimated at the whole-metagenome, with ratio = 1 meaning that *π* in the subsample is equal to *π* in the whole-metagenome. Note that sequencing depth values are in represented logarithmic scale.

### ANIr is robust to low and uneven sequencing depth

To further evaluate if ANIr estimates were also influenced by uneven sequencing depth, we followed the same procedure to that of *π* estimates. Contrasting with *π*, ANIr estimates were remarkably robust to variation in sequencing depth from 5-10X to over 1,400X across all environments and sequencing platforms studied here. Across all subsampled datasets, the differences in ANIr compared to the full dataset remained minimal (diversity ratio ~1; Fig. 1) along with an average absolute error below 0.025% across environments and sequencing platforms (Fig. 2). In fact, discrepancies between subsampled and full-dataset ANIr values, even at 5X depth, were consistently two orders of magnitude smaller than for *π* at the same sequencing depth levels (Figs 1 and 2), supporting previous findings that ANIr remains stable at depth exceeding 10X [14]. As a result, the limited influence of uneven sequencing depth in ANIr estimates makes it a powerful and practical metric for comparative intraspecies diversity studies using metagenomics. This apparent robustness of ANIr likely resided from it being a read-based metric rather than a site-specific estimate, which apparently makes ANIr less prone to stochastic errors introduced -for example- by sequencing.

Despite its benefits under uneven sequencing depth, ANIr also has important limitations. Because ANIr does not capture the site frequency spectrum, it cannot be interpreted in a population genetics framework. This contrasts with widely used site-based measures of intraspecies diversity (e.g. *π*, *θ*, Tajima’s *D*), which provide information on allele frequencies [46–49], allowing to infer underlying evolutionary processes, including selection (positive or balancing), genetic drift, demographic changes (population expansions or bottlenecks), migration, and recombination [46–49]. Additionally, ANIr relies on the availability of a suitable and accurate reference isolate genome or MAG that represents the dominant population genotype [2]. In complex microbial communities with high intraspecies diversity (e.g. soils, oceans), multiple coexisting lineages, or no clear dominant strain, the reference genome may not reflect the true structure of the population. In such cases, ANIr could fail to capture important microdiversity signals or misrepresent the population consensus [2, 14]. As ANIr focuses on consensus identity rather than variant frequency spectra, it may overlook ecologically or clinically relevant rare variants that *π* may capture under sufficient sequencing.

**Figure 5:**
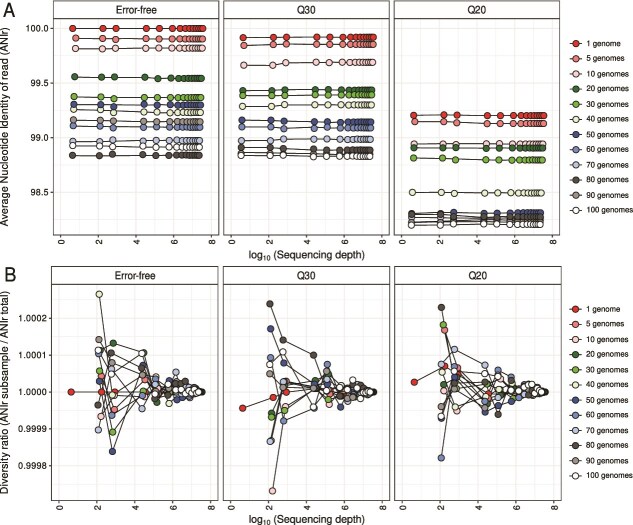
Sequencing errors and uneven sequencing depth impacts ANIr estimates even within a clonal population. (A) Estimation of ANIr values at increasing sequencing depth and isolate diversity on the 32 distinct *in silico* metagenomes with variable sequencing error profiles (i.e. error-free, Q20, Q30). Note that sequencing depth values are in represented logarithmic scale. (B) Estimation of the effect of increasing sequencing depth on the accuracy of ANIr across *in silico* metagenomes with distinct proportions of isolate genomes and sequencing error profiles. The diversity ratio represents the estimated ANIr at each subsample of a metagenome divided by ANIr estimated at the whole-metagenome, with ratio = 1 meaning that ANIr in the subsample is equal to ANIr in the whole-metagenome. Note that sequencing depth values are represented in logarithmic scale.

Nevertheless, the minimal sensitivity of ANIr to sequencing depth and subsequently to sequencing breadth, makes it especially valuable for studies constrained by uneven or limited sequencing depth, as well as for cross-study comparisons where normalizing for *π* is impractical. In these scenarios, ANIr offers a complementary approach for assessing intrapopulation diversity, with strong potential for broad application in both environmental and clinical microbiome research. Further, MAGs recovered from a metagenome usually represent well the natural population (the consensus or average) and thus provide accurate ANIr values for that metagenome and population. In cases that a good reference genome is not available, ANIr can be assessed by overlapping reads that represent the population (identified by mapping at the >95% nucleotide identity against a genome of the species), which alleviates the limitations associated with the use of a reference in the estimation.

### *In silico* metagenomes support trends observed with environmental data

To further validate those findings observed using environmental data, we generated 36 *in silico* metagenomes with 50 million reads each, using a curated set of 100 well-characterized *Sal. ruber* isolates (Supplementary Table S3). These *in silico* metagenomes comprise 1, 5, 10, or up to 100 genomes selected from 100 isolate genomes available (Supplementary Table S4), which shared a range of Average Nucleotide Identity (ANI) of 97.51% to ~100% and an average gene shared fraction of 79.09 ± 2.96% (Supplementary Fig. S9). For each metagenome, we simulated three sequencing error profiles, including reads without polymorphisms (i.e. error-free metagenomes), Q20 (i.e. one error per 1000 bp), and Q30 (i.e. one error per 10 000 bp), resulting in 36 metagenomes (i.e. 12 per error profile) with defined genetic diversity, richness, and error probability.

In the simplest scenario, where only a single genome was used to simulate reads, we observed that *π* across sequencing depth was precisely zero in the error-free metagenome (Fig. 4A; Supplementary Fig. S10). This is expected, as no polymorphisms exist in a truly clonal population, and therefore every position in the alignment is occupied by a unique dominant nucleotide [5]. However, when introducing realistic sequencing error models, simulated at Q20 and Q30 quality thresholds, we observed substantial increases in *π*, reaching values ~0.0075 despite the absence of true biological variation (Fig. 4A; Supplementary Fig. S10). This trend was mirrored in ANIr estimates: in the absence of sequencing errors, ANIr between simulated reads and the reference genome remained at 100% (Fig. 5A; Supplementary Fig. S11). However, for Q20 and Q30 datasets, ANIr decreased to ~99.2% and 99.9% (Fig. 5A; Supplementary Fig. S11), respectively, reflecting the influence of sequencing errors on both metrics.

Using more complex and diverse metagenomes with 5 up to 100 strains, differences among error-free, Q20, and Q30 metagenomes were also observed for *π* (Fig. 4B; Supplementary Fig. S12) and ANIr (Fig. 5B; Supplementary Fig. S13) estimates. These findings indicate that *π* is not only sensitive to true genetic diversity but also to technical bias introduced by base-calling errors, even at high sequencing quality (i.e. Q30; Fig. 4B; Supplementary Fig. S12). Furthermore, given that long-read sequencing technologies have larger sequencing errors per base-pair, with PacBio typically showing 5 errors per 10 000 bp and ONT 100 errors per 10 000 bp, we would expect even larger influences on *π*-based microdiversity estimates. Differences among error-free, Q20, and Q30 metagenomes suggested that sequencing errors could be also a main driver on microdiversity estimate biases in metagenomes and one of the reasons why we did not observe a plateau for *π* with sequencing depth >1400 (Fig. 1).

Focusing on the diversity ratio, meaning differences among subsampled and full-dataset estimates, *π* and ANIr showed the same pattern as observed using environmental data (Fig. 1). Specifically, *π* estimates were underestimated below and above 10X sequencing depth (Fig. 4B; Supplementary Fig. S12), whereas ANIr remained remarkably stable, especially above 10X (5B; Supplementary Fig. S13). Consistently, we also detected that *π* values never plateaued, even at 1800X (Fig. 4B; Supplementary Fig. S12), meaning that differences between subsampled and full datasets persisted, which indicates an underestimation of microdiversity as we hypothesized. Furthermore, we also detected that differences between *π* estimates of subsampled and full datasets were significantly reduced across all simulations between 50X and 200X sequencing depth (Fig. 4), supporting the choice of at least 50X as a suitable target for projected *π* estimates. Given that these trends have been detected using both environmental (Figs 1 and 2) and synthetic (Figs 4 and 5) metagenomes, we strongly considered that current sequencing practices (10X to 100X) may fail to reveal the full extent of intraspecies diversity and to compare genes or species using *π*-based metrics would need to be corrected for sequencing depth, with a minimum sequencing depth of 50X and ideally rarefying to ~200X.

## Concluding remarks

Here, we showed that uneven sequencing depth (and sequencing breadth) significantly biased *π* estimates below 50X, potentially resulting in inaccurate estimations of intraspecies diversity when analyzing microdiversity below that sequencing depth threshold. To solve this, we suggest to: (i) sequence metagenomes with larger sequencing depth to increase the fraction of the intraspecies diversity that is covered in each species, (ii) apply rarefying-based approaches of sequencing depth to fixed thresholds above 50X and ideally to 200X, when aiming to estimate *π* at gene and/or genome level, and (iii) use the ANIr metric as a complementary metric for estimating microdiversity without needing rarefying sequencing depth, allowing the comparison among samples and species with uneven and/or low sequencing depth. In summary, we expect that the recommendations provided here will help minimize biases in comparative microdiversity studies using metagenomics, thereby facilitating the generation of quantitative, standardized, and meaningful results on both clinical and environmental settings.

## Supplementary Material

ISMECOMMUN-D-24-00435R2_Supp_Material_ycaf228

ISMECOMMUN-D-24-00435R2_Supp_Tables_ycaf228

## Data Availability

*The datasets analyzed during the current study are available in the European Nucleotide Archive (ENA) repository, at*  https://www.ebi.ac.uk/ena/browser/home  *under BioProject accession numbers* PRJEB75750, PRJEB52999, and PRJNA763692. Custom R code developed in this study for the estimation of average error (%) is available at https://github.com/ebustos128/Uneven-sequencing-can-bias-estimates-of-microbial-intraspecies-diversity.
