# Supplementary material for: Uneven sequencing (coverage) depth can bias microbial intraspecies diversity estimates and how to account for it
Source: ISME Commun. 2025 Dec 6;5(1):ycaf228. doi: 10.1093/ismeco/ycaf228 (PMC12753299; doi:10.1093/ismeco/ycaf228)

**Uneven sequencing (coverage) depth can bias microbial intraspecies diversity estimates and how to account for it**

Esteban Bustos-Caparros^1^*, Tomeu Viver^1^, Juan F. Gago^1^, Stephanus N. Venter^2^, Rafael Bosch^3^, Konstantinos T. Konstantinidis^4^, Luis M. Rodriguez-R^5,6^*, Ramon Rossello-Mora^1^*.

^1^ Marine Microbiology Group (MMG), Department of Animal and Microbial Biodiversity, Mediterranean Institute for Advanced Studies (IMEDEA, CSIC-UIB), Esporles, Spain.

^2^ Department of Biochemistry, Genetics and Microbiology, and Forestry and Agricultural Biotechnology Institute (FABI), University of Pretoria, Pretoria, South Africa.

^3^ Microbiologia, Departament de Biologia, Edifici Guillem Colom, Universitat de les Illes Balears, Campus UIB, 07122 Palma de Mallorca, Spain.

^4^ School of Civil and Environmental Engineering and School of Biological Sciences, Georgia Institute of Technology, Atlanta, GA, USA.

^5^ Department of Chemistry and Biosciences, Aalborg University, Aalborg, Denmark.

^6^ Department of Microbiology and Digital Science Center (DiSC), University of Innsbruck, Innsbruck, Austria.

*Corresponding authors: Esteban Bustos-Caparros, Marine Microbiology Group (MMG), Department of Animal and Microbial Biodiversity, Mediterranean Institute for Advanced Studies (IMEDEA, CSIC-UIB), Carrer Miquel Marquès 21, Esporles, Illes Balears, 07190, Spain. Email: [ebustos@imedea.uib-csic.es](mailto:ebustos@imedea.uib-csic.es), Luis M. Rodriguez-R, Department of Chemistry and Biosciences, Aalborg University, Frederik Bajers Vej 7H, 9000 Aalborg, Denmark. Email: [lmrodriguezr@gmail.co](mailto:lmrodriguezr@gmail.com)m, Ramon Rossello-Mora, Marine Microbiology Group (MMG), Department of Animal and Microbial Biodiversity, Mediterranean Institute for Advanced Studies (IMEDEA, CSIC-UIB), Carrer Miquel Marquès 21, Esporles, Illes Balears, 07190, Spain. Email: ramon@imedea.uib-csic.es

**Running title:** Sequencing depth can bias microdiversity

**Supplementary Figure S1:** Schematic representation of the methods used to evaluate the sequencing (coverage) depth bias on microdiversity estimates (pi and ANIr) using both environmental and synthetic metagenomes.


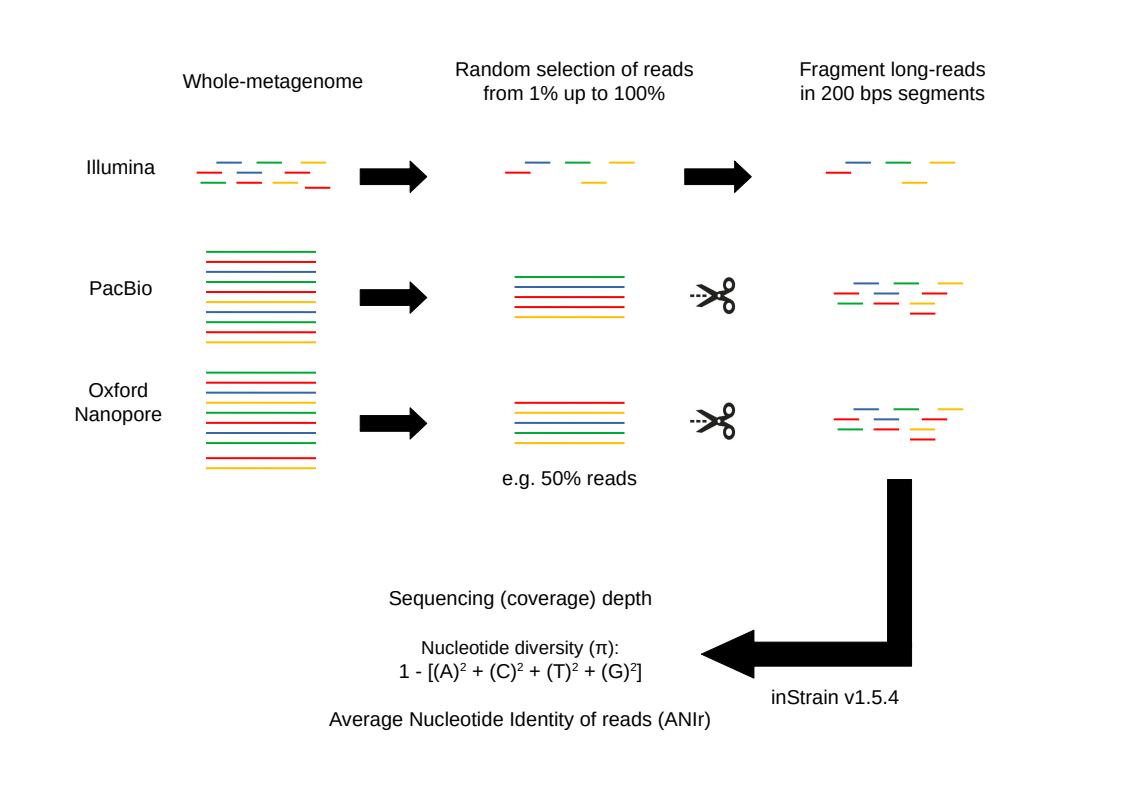


**Supplementary Figure S2:** Model plot representing the microdiversity estimate (nucleotide diversity or ANIr) trend along an increasing sequencing (coverage) depth. Each line represents the microdiversity estimate (y-axis), represented as Microdiversity ratio, and sequencing (coverage) depth (x-axis) obtained for a single MAG across each metagenome. The Microdiversity ratio was calculated by dividing the nucleotide diversity or ANIr obtained from each fraction of a metagenome by the nucleotide diversity or ANIr obtained from the entire metagenome (100% of reads). Green lines indicate positive correlations (i.e. increasing microdiversity with coverage depth), and red lines indicate negative correlations (i.e. decreasing microdiversity with coverage depth).

​
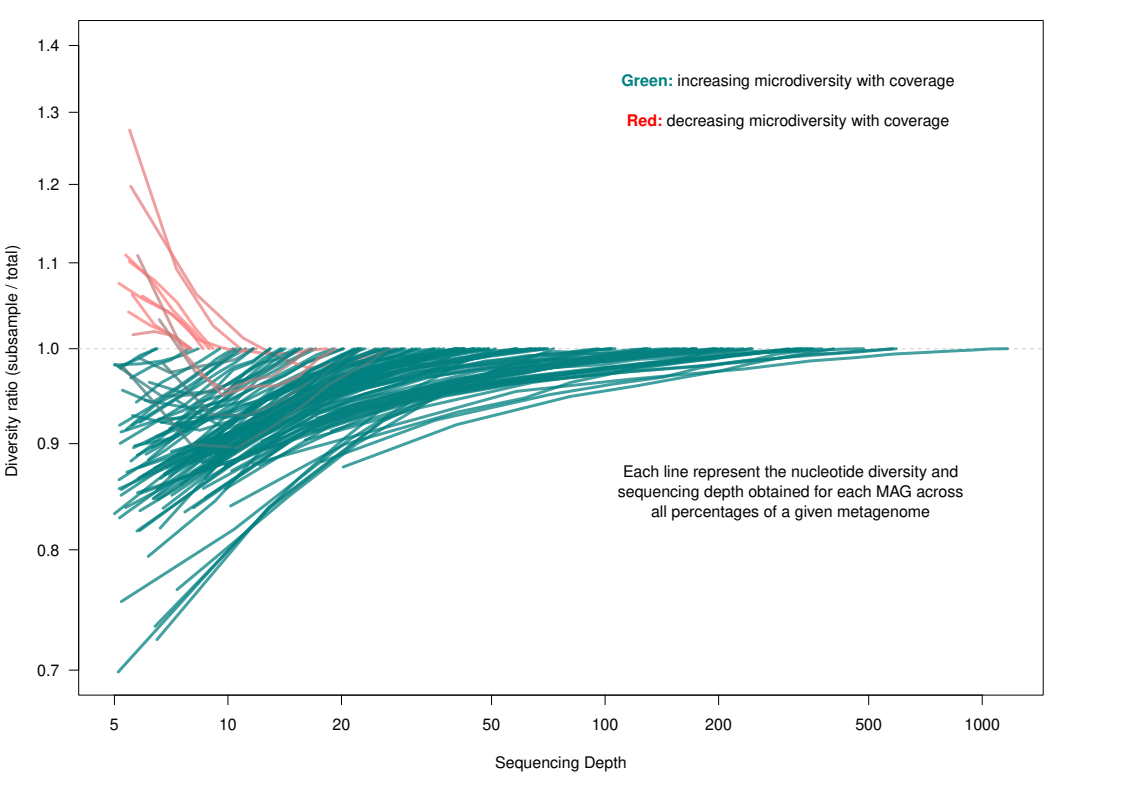
​

**Supplementary Figure S3:** Estimation of the impact of sequencing depth from 500X to ~1,400X on the accuracy of nucleotide diversity (*π*) estimates across hypersaline and marine metagenomes using Illumina sequencing platform. The microdiversity ratio represents the *π* obtained for each subsample of a metagenome divided by the *π* of the whole-metagenome, with ratio = 1 meaning that microdiversity in the subsample is equal to microdiversity in the whole-metagenome. Note that each line represents the microdiversity estimate of one MAG in a given metagenome. Green lines indicated larger microdiversity at increasing sequencing depth (i.e. expected trend; microdiversity ratio < 1).

​
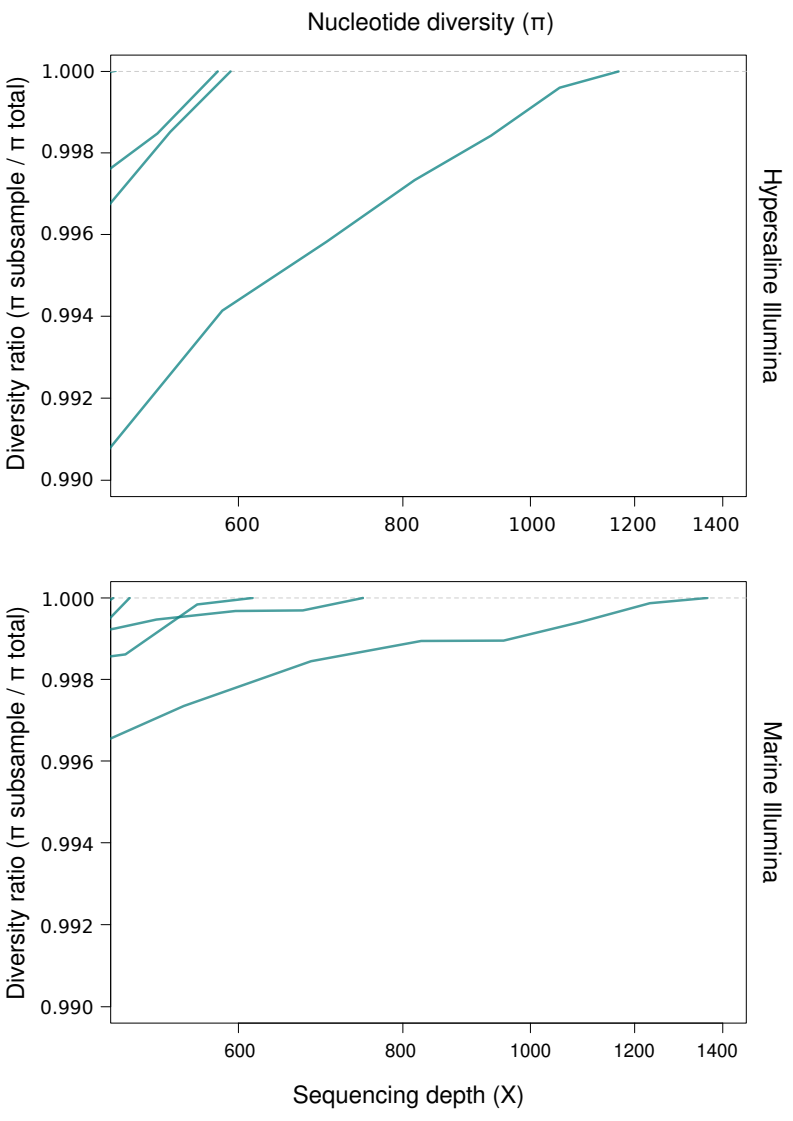
​

**Supplementary Figure S4:** Barplots showing nucleotide diversity (π) estimates of each of the ten species analyzed from the hypersaline microbiome using Illumina metagenomes (n = 15). Color gradient represents the sequencing depth values across metagenomes per each species.


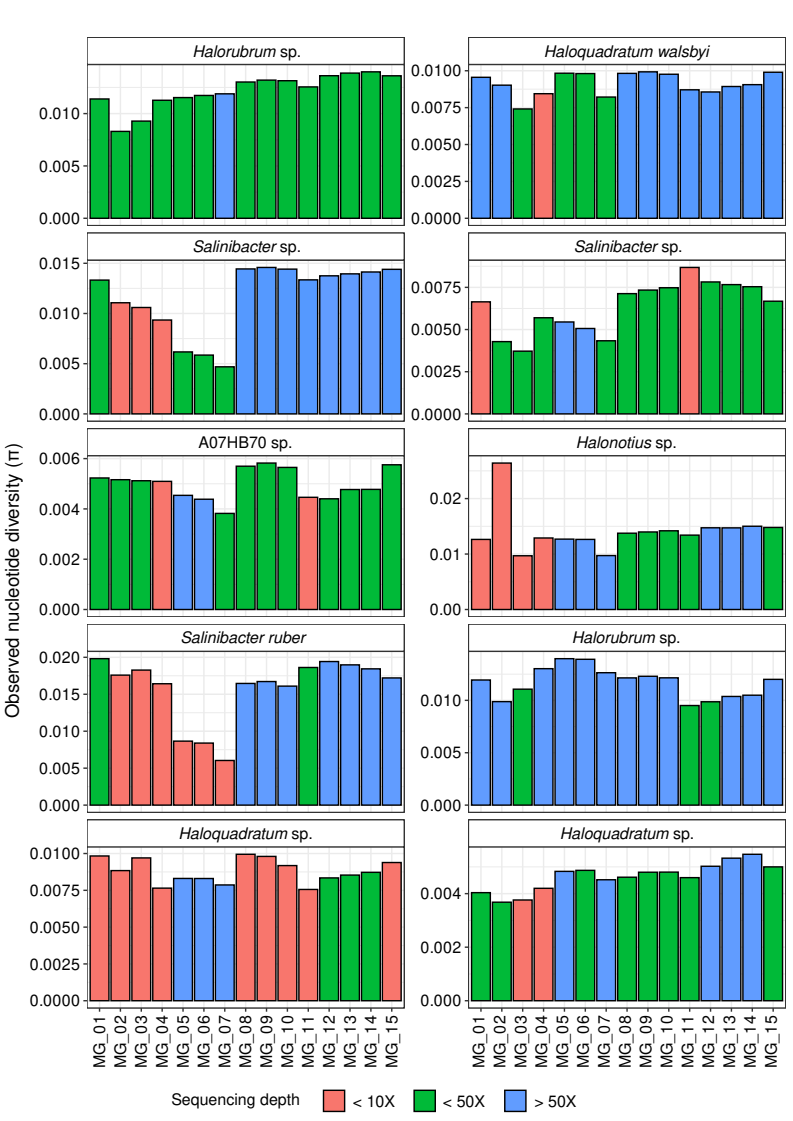


**Supplementary Figure S5:** Barplots showing nucleotide diversity (π) estimates of each of the ten species analyzed from the marine microbiome using PacBio metagenomes (n = 15). Color gradient represents the sequencing depth values across metagenomes per each species.


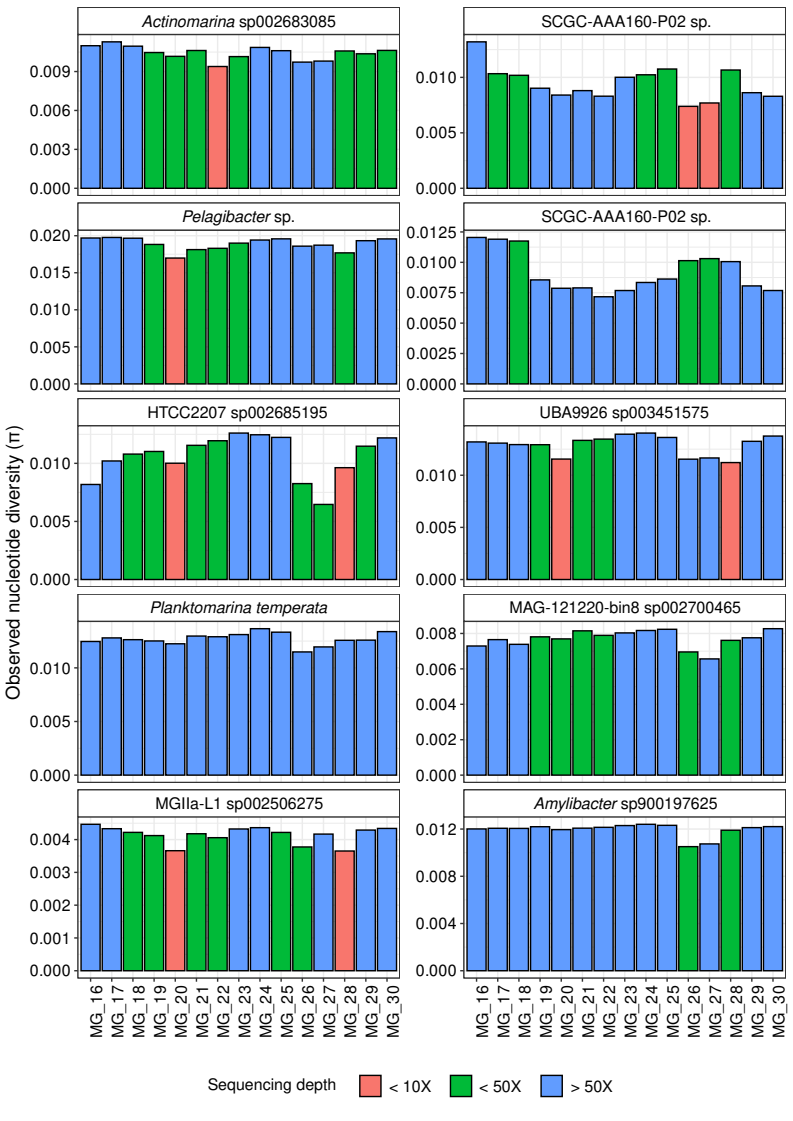


**Supplementary Figure S6:** Barplots showing nucleotide diversity (π) estimates of each of the ten species analyzed from the marine microbiome using Illumina metagenomes (n = 4). Color gradient represents the sequencing depth values across metagenomes per each species.


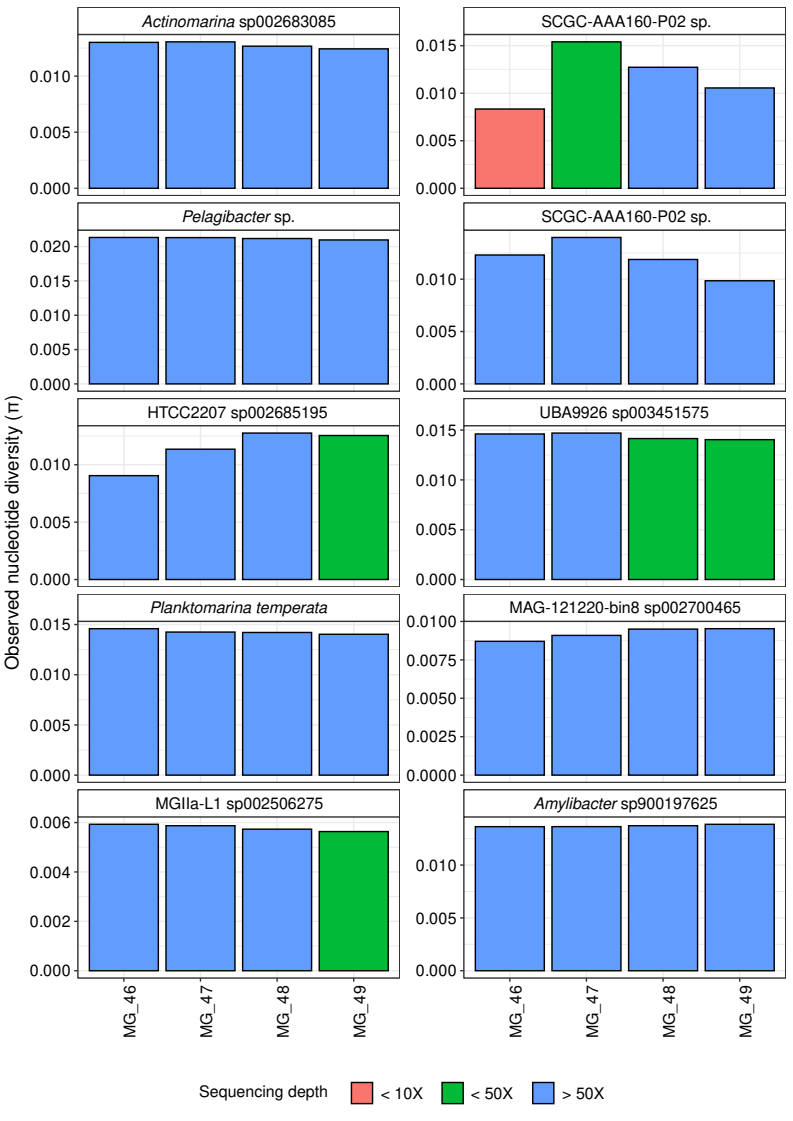


**Supplementary Figure S7:** Barplots showing nucleotide diversity (π) estimates of each of the ten species analyzed from the human gut microbiome using ONT metagenomes(n = 15). Color gradient represents the sequencing depth values across metagenomes per each species.


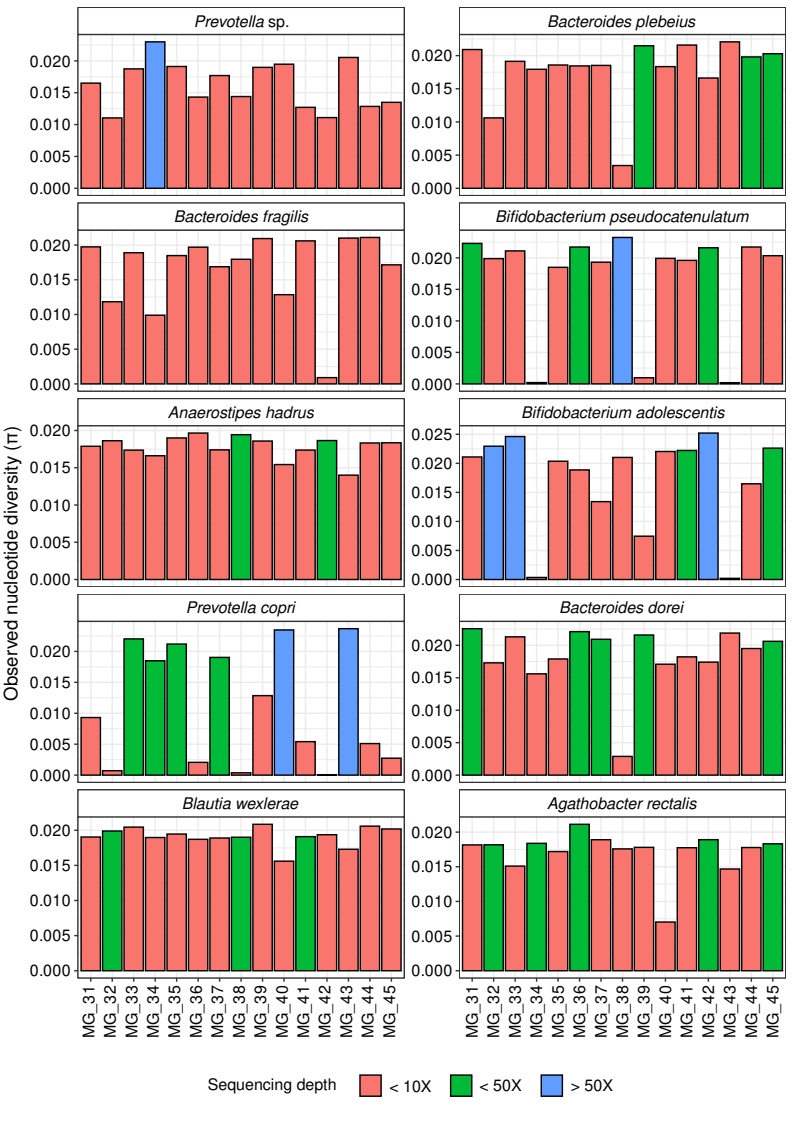


**Supplementary Figure S8:** Barplots showing nucleotide diversity (π) estimates of each of the ten species analyzed from the human gut microbiome using Illumina metagenomes (n = 4). Color gradient represents the sequencing depth values across metagenomes per each species.


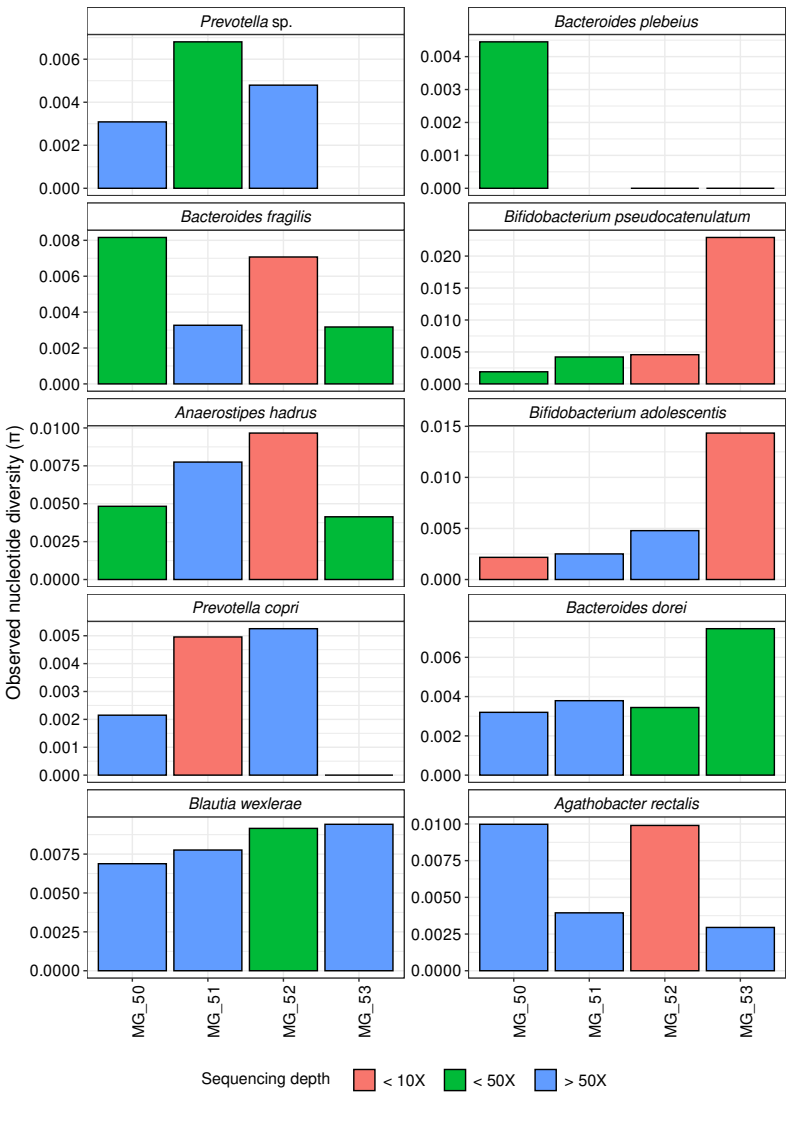


**Supplementary Figure S9:** Average Nucleotide Identity (ANI) and shared genome fraction (%) comparison among the 100 isolate genomes of *Salinibacter ruber*.


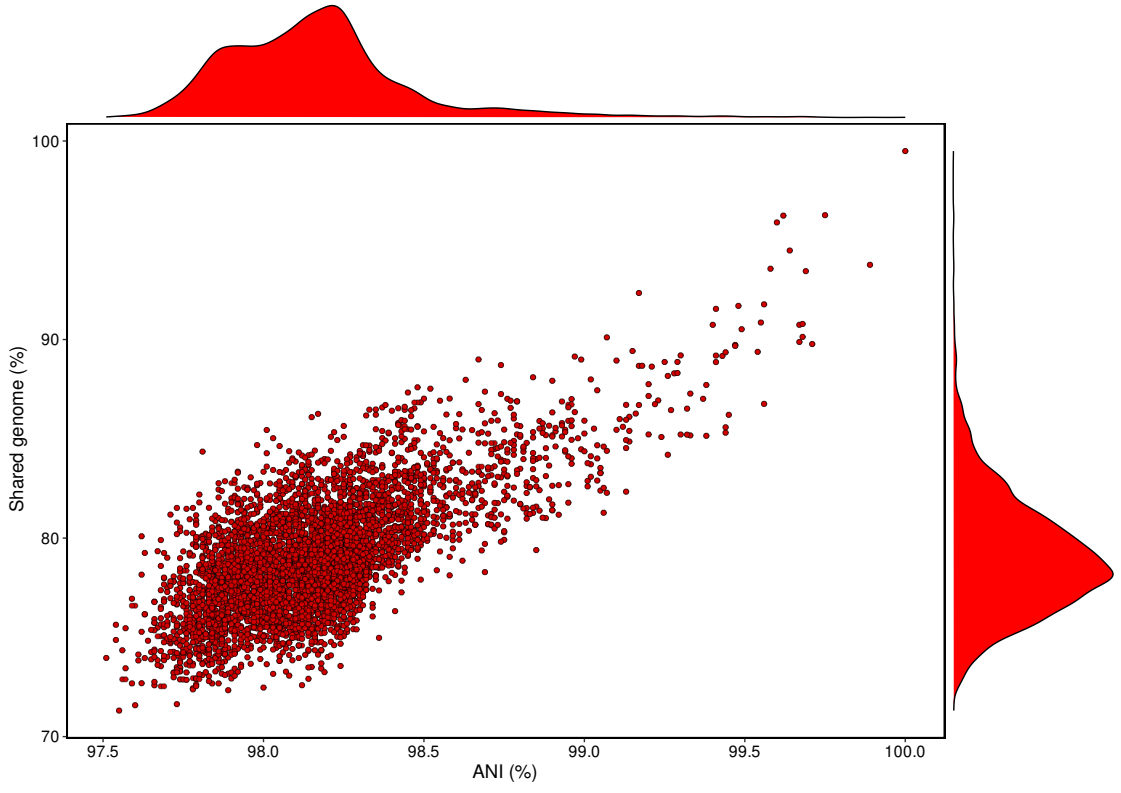


**Supplementary Figure S10:** Estimation of nucleotide diversity (π) values at increasing sequencing depth and isolate diversity on the 32 distinct *in silico* metagenomes with variable sequencing error profiles.


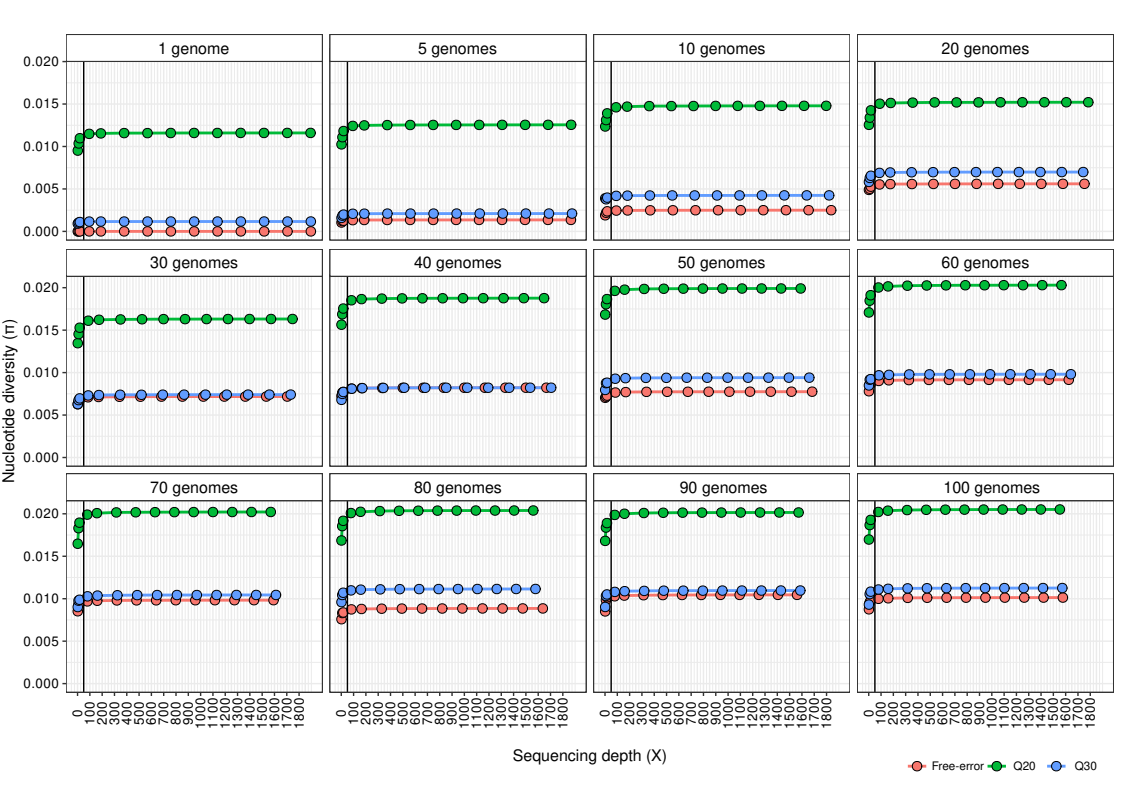


**Supplementary Figure S11:** Estimation of ANIr values at increasing sequencing depth and isolate diversity on the 32 distinct *in silico* metagenomes with variable sequencing error profiles.


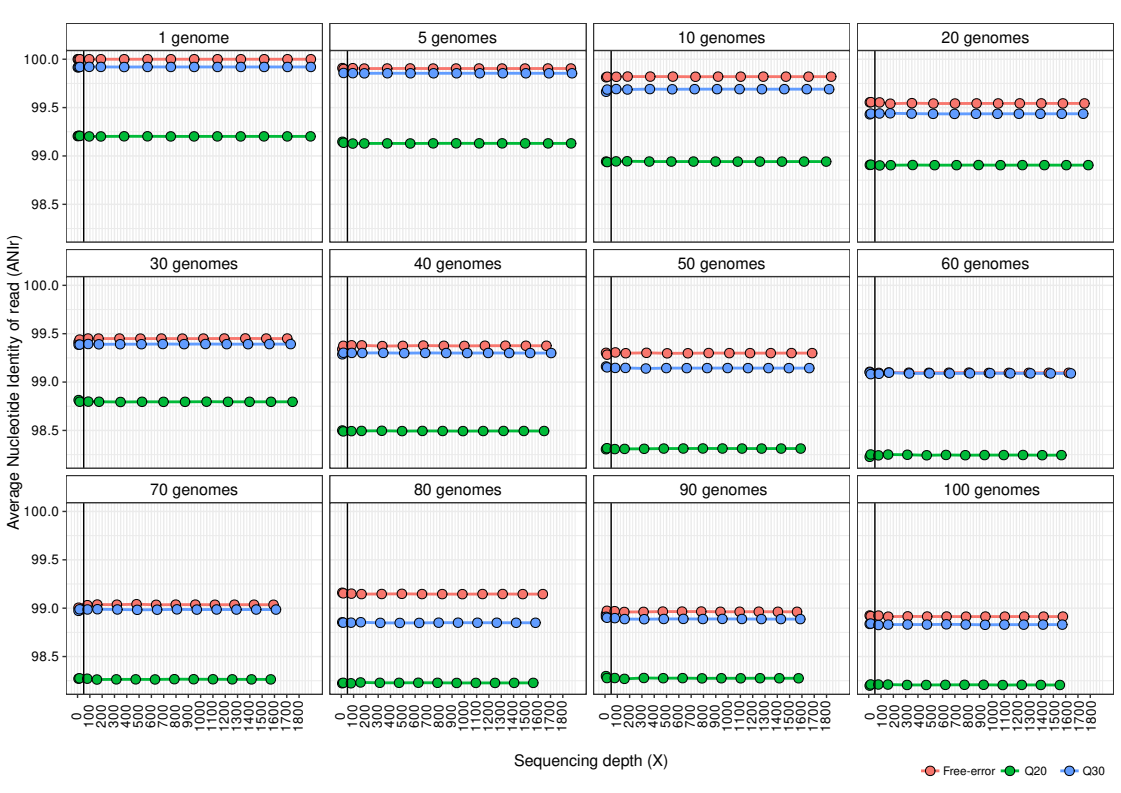


**Supplementary Figure S12:** Estimation of the effect of increasing sequencing depth on the accuracy of nucleotide diversity (π) across *in silico* metagenomes with distinct proportions of isolate genomes and sequencing error profiles. The diversity ratio represents the estimated π at each subsample of a metagenome divided by π estimated at the whole-metagenome, with ratio = 1 meaning that π in the subsample is equal to π in the whole-metagenome. Note that we showed the trends up to 400X to better show the effects of sequencing depth on nucleotide diversity (π) estimates. Lines indicate a sequencing depth of 50X and 200X.


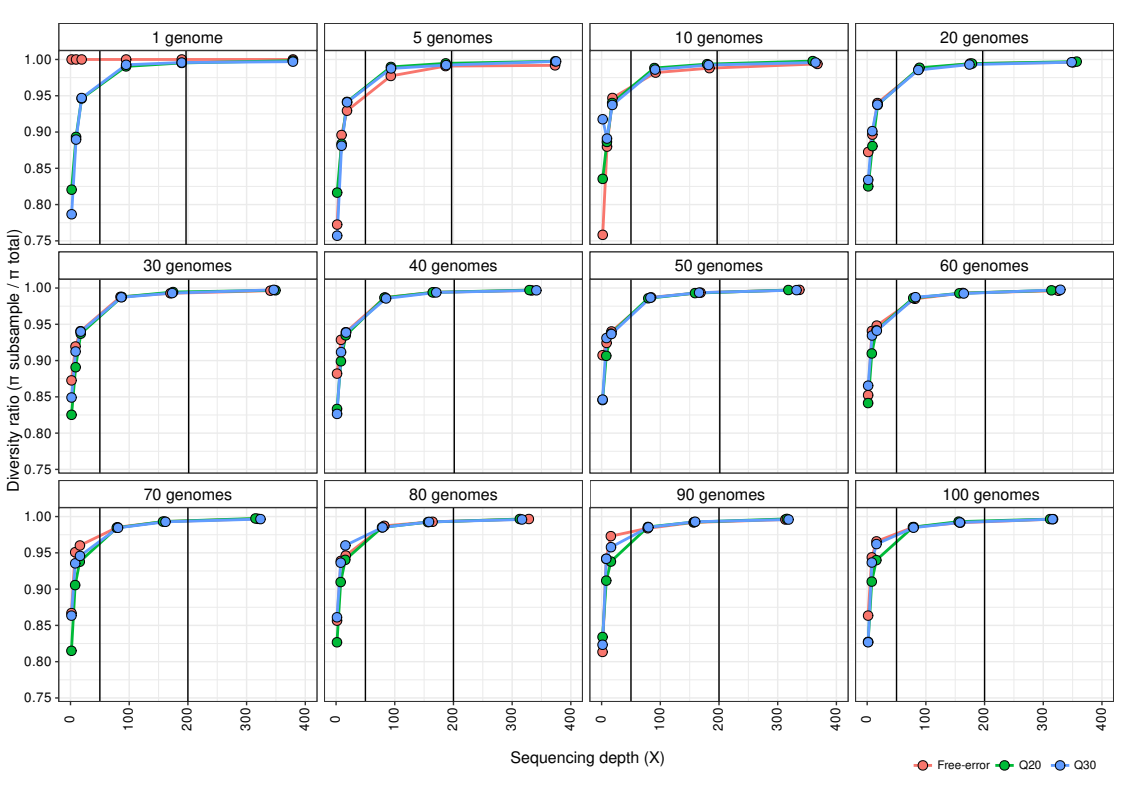


**Supplementary Figure S13:** Estimation of the effect of increasing sequencing depth on the accuracy of ANIr across *in silico* metagenomes with distinct proportions of isolate genomes and sequencing error profiles. The diversity ratio represents the estimated ANIr at each subsample of a metagenome divided by ANIr estimated at the whole-metagenome, with ratio = 1 meaning that ANIr in the subsample is equal to ANIr in the whole-metagenome. Note that we showed the trends up to 400X to better show the effects of sequencing depth on ANIr estimates. Lines indicate a sequencing depth of 50X and 200X.


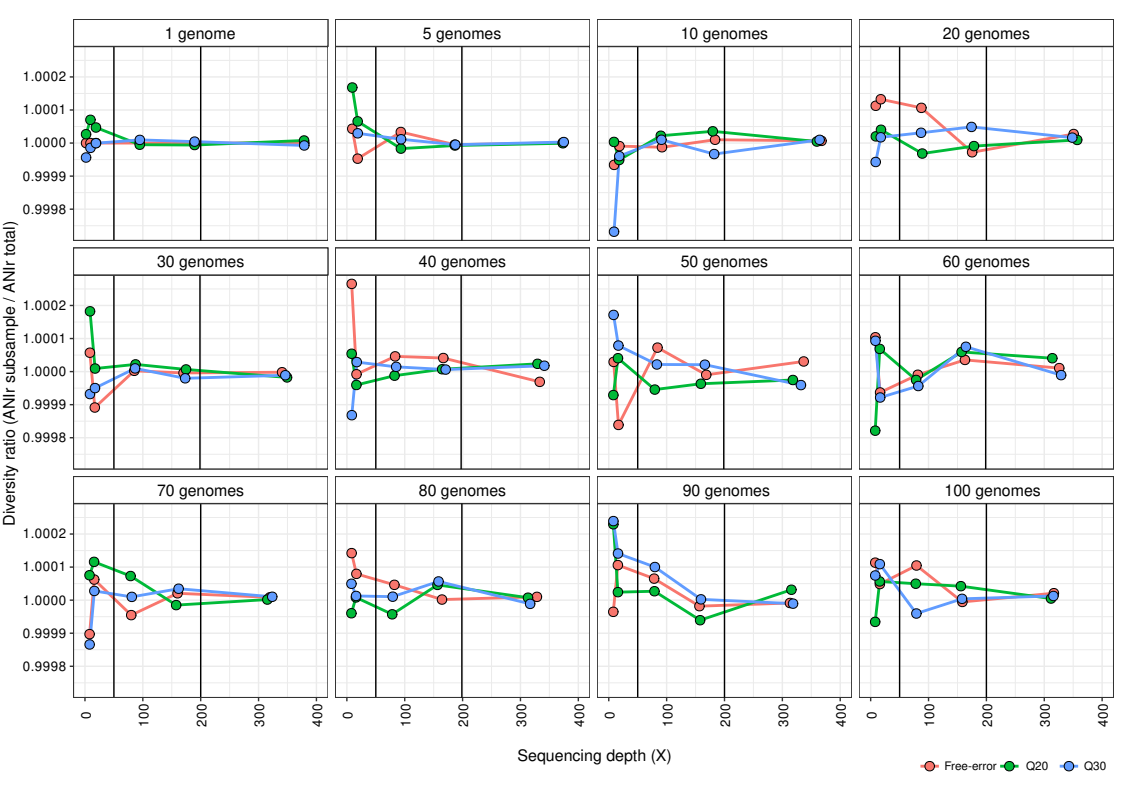

Supplement: ISMECOMMUN-D-24-00435R2_Supp_Material_ycaf228 [file ismecommun-d-24-00435r2_supp_material_ycaf228.docx]
